# Inhibition of Cathepsin B by E-64 Induces Oxidative Stress and Apoptosis in Filarial Parasite

**DOI:** 10.1371/journal.pone.0093161

**Published:** 2014-03-25

**Authors:** Mohit Wadhawan, Neetu Singh, Sushma Rathaur

**Affiliations:** Department of Biochemistry, Faculty of Science, Banaras Hindu University, Varanasi, India; New England Biolabs, United States of America

## Abstract

**Background:**

Current available antifilarial drug strategies only eliminate the larval stages of filarial parasites. Therefore, there is an urgent need of drugs which are macrofilaricidals. Identification of molecular targets crucial for survival of parasite is a prerequisite for drug designing. Cathepsin B, a cysteine protease family member is known to play crucial role in the normal growth, digestion of nutrients, exsheathment of the helminth parasites. Therefore, we targeted this enzyme in the filarial parasite using its specific inhibitor, E-64.

**Methods and Findings:**

We have exposed the parasites to E-64 and observed their motility and viability at various time intervals. It caused marked decrease in the motility and viability of the parasites ultimately leading to their death after 8 hours. It is well known that E-64 protects the cell from apoptosis, however, it causes apoptotic effect in carcinoma cell lines. To understand the mechanism of action of E-64 on parasite survival, we have measured levels of different apoptotic markers in the treated parasites. E-64 significantly reduced the level of ced-9 and activity of tyrosine phosphatases, cytochrome c oxidase. It also activated ced-3, homolog of mammalian caspase 3 suggesting initiation of an apoptotic like event in the filarial parasites. Different antioxidant enzymes were also evaluated to further explore the mechanism behind the death of the parasites. There was marked decrease in the level of GSH and activity of Glutathione reductase and glutathione-s-transferase leading to increased generation of reactive oxygen species. This led to the induced oxidation of fatty acids and protein which might alter the mitochondrial membrane permeability.

**Conclusion:**

This study suggests that inhibition of cathepsin B by E-64 generates oxidative stress followed by mitochondrial mediated apoptotic like event in filarial parasites leading to their death. Hence, suggesting filarial cathepsin B as a potential chemotherapeutic target for lymphatic filariasis.

## Introduction

Lymphatic filariasis is a mosquito-borne, chronically disabling parasitic disease caused by tissue dwelling filarial nematodes: *Wuchereria bancrofti*, *Brugia malayi* and *Brugia timori*. This tropical disease is considered as a major obstacle to socioeconomic development in the endemic countries [1]. It is identified as the second leading cause of permanent and long-term disability, afflicting over 120 million people in 81 countries worldwide and an estimated 1.34 billion at the risk of infection [2]. In 1997, the World Health Organization (WHO) launched a global programme to eliminate lymphatic filariasis by conducting mass drug administration in the affected areas [3]. Annual treatment of the disease includes available antifilarial drugs like diethylcarbamazine (DEC), albendazole and ivermectin, administered either alone or in combination. These drug treatments effectively eliminate the larval stages but not the adult worms. Moreover, discovery of new anti-filarial drugs has been hampered due to the dynamics of filariasis transmission and long life span of the adult worms in the host. Thus, search for broad range of macrofilaricidal agents remain an urgent need. It has been suggested that the enzyme inhibitors and their analogues can be used as drug by selecting target enzymes or metabolic pathways which are crucial for the survival and persistence of the parasites [4].

Earlier reports from our laboratory have shown the presence of a cathepsin B like cysteine protease activity in the adult and larval stages of *Setaria cervi* (*S. cervi*), a bovine filarial parasite [5]. This parasite resembles *W. bancrofti*, a human filarial parasite in its nocturnal periodicity and antigenic cross reactivity [6]. These cathepsins are thought to be the most prevalent of all the other cathepsins in nematodes [7], [8]. They play a potential role in the normal growth, digestion of haemoglobin and other nutrients, thereby maintaining normal metabolism of the helminth parasites [9], [10]. It has been reported earlier that downregulation of cathepsin B can induce apoptosis by suppressing Bcl2 expression and activating BAX accompanied by alternation in mitochondrial membrane potential in human glioma cells [11]. Therefore, we have used L-trans-Epoxysuccinyl-leucylamido (4-guanidino)butane (E-64), a specific inhibitor of cathepsin B, H and L that irreversibly binds to an active thiol group in many cysteine proteases through its trans-epoxysuccinyl group to form a thioether linkage [12], [13] to inhibit cathepsin B in *S. cervi* and observe its effect on parasite survival. E-64 is one of the most suitable cysteine protease inhibitors used *in vivo* because of its effective permeability into cells and tissues and low toxicity [14].

Here, in our study we have measured different apoptotic markers *viz.* ced-9, ced-3/caspase 3, Cytochrome C oxidase, and protein tyrosine phosphatases in the control as well as treated parasites. Earlier studies in our laboratory have shown that the antifilarial drug, DEC, in combination with aspirin induced oxidative stress leading to mitochondria mediated apoptosis in *S. cervi* [15]. Several reports also show that the death receptor-associated extrinsic pathway as well as mitochondria-dependent intrinsic pathway of apoptosis are triggered by oxidative damage [16], [17]. Therefore, we have also evaluated different oxidative stress parameters like reactive oxygen species generation, lipid peroxidation, protein carbonyls and NADPH oxidase too. The levels of various enzymatic and non-enzymatic antioxidants were observed as these parasites possess a strong anti-oxidant system to evade from the harsh host environment [18]. The mechanism of action of E-64 in survival of filarial parasites and possibility of use of filarial cathepsin B as a drug target for lymphatic filariasis has also been studied.

## Materials and Methods

### Ethics statement

No experimental animals and humans/human samples were used in this study. The Indian water buffaloes harbouring *Setaria cervi* worms are slaughtered in the Slaughter House, Orderly bazaar, Varanasi, Uttar Pradesh, India for table purpose and no animals were slaughtered specifically for our study. The *S. cervi* worms are the waste material for the slaughter house and were procured from the peritoneal folds of freshly slaughtered animals. They were purchased by us and brought to the laboratory.

### Chemicals and reagents

E-64, Caspase-3 substrate, 4-(Hydroxymercuri)benzoic acid sodium salt (PHMB), Z-Ala-Arg-Arg-4 MβNA, cytochrome c, Ortho phospho-L-tyrosine were purchased from Sigma Aldrich chemical company, USA. All other chemicals were purchased from Hi-Media Laboratories, Mumbai, India.

### Preparation of extract

The adult female motile *S. cervi* worms were procured from the peritoneal folds of freshly slaughtered animals and brought to the laboratory in Kreb's Ringer bicarbonate (KRB) buffer bicarbonate (KRB) buffer supplemented with streptomycin, penicillin, glutamine and 1% glucose (maintenance medium). The worms were thoroughly washed with phosphate buffered saline (PBS), pH 7.0. A 10% w/v homogenate of adult parasites was prepared as per the standardised procedure of our laboratory [19]. Briefly, worms were homogenized in 50 mM phosphate buffer, pH 7.0 using motor driven REMI homogenizer (RQ127A) at 4°C. The extract was centrifuged at 5000 g followed by 15,000 g for 30 min. The clear supernatant thus obtained was stored at −20°C for further use.

### Exposure of worms to E-64

Equal number (n = 10) of adult female *S. cervi* were incubated in the 20 ml KRB maintenance medium containing different concentrations of E-64 (10 μM, 20 μM and 40 μM final concentration) for 8 h at 37°C and 5% CO_2_. The worms incubated in the maintenance medium only served as control. After 8 h worms were recovered and washed thoroughly with PBS, homogenized and assayed for various enzyme activities.

### Effect on parasite motility

The motility of parasites was performed by visual inspection. The parasites incubated in E-64 containing medium were assessed visually till 8 h and scored either positive or negative (+/-) depending on their motility. After 8 h the recovery of motility was recorded by keeping the worms in fresh medium (devoid of E-64) for 1 h. Parasite motility was scored as -, no movement; +, least active; ++, less active; +++, moderately active; and ++++, highly active. To check the effect of E-64 on microfilariae (mf), adult female parasites were dissected longitudinally and released mf were collected in KRB maintenance medium and visualized under microscope at 40× (Motic B1 series).

### Effect on parasite viability

The MTT assay was carried out to determine parasite viability according to the method of Mosmann *et al* 1988 with slight modifications [20]. The treated worms were incubated in phosphate buffered saline (PBS) containing 1.0 ml of 0.5 mg/ml MTT [3-(4, 5-dimethylthiazol-2yl)-2, 5-diphenyl tetrazolium bromide] for 2 h at 37°C. The worms were then transferred to a fresh eppendorf containing 200 μl of dimethyl sulphoxide (DMSO) to solubilize the formazan crystals. After 1 h the medium was then carefully removed without disturbing the dark blue formazan crystals. The optical density of the resulting formazan solution was determined on a microplate reader (BioRad) at a wavelength of 540 nm. The half maximal effective concentration (EC_50_) of E-64 was calculated by plotting the graph between the concentration of E-64 and % viability of the adult parasites after 8 h of treatment. Here, EC_50_ refers to the concentration of the inhibitor where 50% of its maximal effect is observed or 50% of the parasite viability was reduced after specified incubation duration.

### Assay for cathepsin B cysteine proteases

The Cathepsin Β activity was determined by the colorimetric method of Barrett (1977) using Z-Arg-Arg-4mβNA as substrate with slight modifications [21]. The crude extract was pre-incubated at 37°C for 5 min in 50 mM sodium acetate buffer, pH 5.0 containing 1 mM EDTA and 5 mM DTT. The substrate (final concentration, 100 μM) was added to make the final assay volume of 1.0 ml. The reaction mixture was incubated at 37°C for 30 min. The reaction was terminated by adding equal volume of stopping reagent containing Fast Garnet GBC salt (1 mg/ml), 10 mM pHMB and 50 mM EDTA, pH 6.0. The extraction of product, β-napthylamine (β-NA), was carried out with n-butanol. After complete layer separation, the absorbance was measured in n-butanol layer and activity was calculated using molar extinction coefficient of β-napthylamine solution as 31.5 M^−1^cm^−1^sec^−1^ at 520 nm. One unit of enzyme activity was defined as the amount of enzyme liberating 1 μmol of βNA per minute at 37°C. The half maximal inhibitory concentration (IC_50_) was calculated by plotting the graph between the different concentration of E-64 and the % inhibition in cathepsin B activity. Here, IC_50_ indicates the concentration of the E-64 required to inhibit the parasitic cathepsin B activity by half.

### Caspase 3 activity assay

The activity of caspase-3-like proteases in the extract was determined using a colorimetric caspase-3 (Sigma) assay with minor modifications [22]. The assay was carried out in a 96 well plate. Briefly, a 100 μl reaction mixture containing 30 μl of adult worm extract and 10 μl of the caspase-3 substrate acetyl-Asp-Glu-Val-Asp-p-nitroanilide (final concentration, 200 μM) in assay buffer was incubated for 90 min at 37°C. The absorbance of the yellow colored product formed was taken at 405 nm using a microtiter plate reader. One unit of the caspase 3 activity was defined as the μmols of p-nitroanilide released per min per ml at 37°C.

### Expression analysis of ced-3 and ced-9 genes by RT-PCR

Total RNA was isolated using Trizol reagent according to the laboratory standardized protocol and was reverse-transcribed into single stranded cDNA with Moloney murine leukemia virus-reverse transcriptase and oligo (dT) primer. The ced-3 and ced-9 genes were amplified using specific primers according to Prasanta Saini *et al* [23]. The following conditions were used for the PCR reaction: 1×PCR buffer mix, 10 mM dNTP mix, 5 pmol of each primer, and 1 unit of Taq DNA polymerase in a total volume of 25 μl. cDNA was denatured at 95°C for 2 min, annealed at 47°C for 1 min, and elongated at 72°C for 3 min for 35 cycles. PCR products were separated on a 1% agarose gel, and photographs were taken using gel documentation system (BioRad). GAPDH was used as the loading control.

### Assay for cytochrome c oxidase (COX) activity

The Cytochrome c oxidase assay is based on the oxidation of ferrocytochrome c to ferricytochrome c in the presence of COX and observing the decrease in absorbance at 550 nm [24]. For enzyme assay, the mitochondria was isolated from the parasites according to the method of Ericson NG et al, 2012 with minor modifications [25]. Briefly, 10% adult female extract was prepared in homogenization buffer containing 0.32 M sucrose, 1 mM EDTA, 10 mM Tris-HCl, pH 7.8. The homogenate was then centrifuged at 1000 g for 10 min at 4°C to pellet down the nuclei and cell debris. The supernatant was collected and again centrifuged at 1000 g for 10 min at 4°C to remove the remaining nuclear material. The supernatant was taken and centrifuged at 13000 g for 20 min to obtain the mitochondrial pellet.

The Ferrocytochrome c (substrate) was prepared by adding 10 μl of 50 mM DTT solution to cytochrome c solution (10 μM). The solution was mixed well and incubated for 20 minutes at RT. The reaction was started by adding 50 μl substrate solution to the reaction mixture containing 50 mM Tris buffer, pH 8.0, 10 μl NP-40, mitochondrial extract (0.5–2 μg) in a final reaction volume of 100 μl. The change in absorbance was read continuously at regular interval of 30 seconds for 2 mins at 550 nm. The activity of COX was calculated using the extinction coefficient of the reduced cytochrome c solution (19.6 mM^−1^ cm^−1^) at 550 nm.

### Assay of tyrosine phosphatases

The Tyrosine phosphatase activity was assayed using the specific substrate O-P-L-Tyrosine by measuring the free phosphate liberated [26]. The enzyme was incubated at 40°C in 1 ml of a solution containing 10 mM substrate, 50 mM sodium acetate (pH 5.0) and 100 mM NaCl. The reaction was stopped after 20 min by addition of 1 ml of 3% ammonium molybdate in 200 mM sodium acetate pH 4.0 followed by further addition of 0.1 ml of 1% ascorbic acid in 200 mM sodium acetate pH 4.0. The color was allowed to develop for 30 min. The free phosphate liberated was measured by the quantitation of reduced phosphomolybdic acid at 700 nm using a molar extinction coefficient of 4×10^3^ M^−1^cm^−1^.

### Measurement of Glutathione level

The GSH level was measured according to the method of Ellman with minor modifications [27]. Equal volumes of 5% meta-phosphoric acid and the adult worm extract were mixed and centrifuged at 3000 rpm for 10 min at 4°C. The total 2 ml reaction mixture containing 100 μl of supernatant, 1.88 ml 0.1 M potassium phosphate buffer, pH 8.0 and 0.02 ml of 4% DTNB [5,5′-dithiobis-(2-nitrobenzoic acid)] was incubated at room temperature (RT) for 15 min. The absorbance of color developed was recorded at 412 nm. The level of GSH was calculated from the standard graph prepared using GSH.

### In vitro effect of E-64 on GSH

To see the effect of E-64 on GSH, 10 μM GSH was incubated with different concentration of E-64 (5, 10, 20, 40 μM) for 20 minutes at 37°C prior to the addition of DTNB. A set without E-64 was taken as the control. Further, 1.88 ml 0.1 M potassium phosphate buffer, pH 8.0 and 0.02 ml of 4% DTNB was added to make the reaction volume upto 2 ml and incubated at RT for 15 min for color development. The absorbance of color developed was recorded at 412 nm.

### Assay of total Reactive oxygen species (ROS) generation

The total reactive oxygen species (ROS) production was measured using colorimetric assay. The method relies on conversion of Nitro blue tetrazolium (NBT) into blue formazan crystals in the presence of the superoxide anion. The assay was performed in inhibitor treated and control worms by following the method as described by Choi et al. with slight modifications [28]. Briefly, the *S. cervi* worms were incubated with 2% NBT solution for 1 h at RT. After incubation, worms were washed with 10 mM phosphate buffer saline followed by wash with methanol. Then the worms were suspended in 2 M KOH to disrupt the cell membrane and then in DMSO to dissolve the formazan crystals with gentle shaking for 10 min at RT. The absorbance was read at 620 nm.

### Glutathione reductase (GR) activity

The GR activity was assayed according to the method described by Carlberg and Mannervik [29]. The reaction was initiated by the addition of 0.1 mM NADPH to the mixture of enzyme in 50 mM potassium phosphate buffer pH 7.0 containing 2 mM EDTA and 0.5 mM GSSG. The change in O.D. was monitored at 340 nm for 3 min by a UV–Vis spectrophotometer. One unit of GR activity is defined as the amount of enzyme that catalyzes the reduction of 1 μmol of NADPH per minute (ε_340 nm_ for NADPH 6.22 mM^−1^ cm^−1^).

### Glutathione-s-transferase (GST) activity

The GST activity in crude extract was estimated according to the method of Habig using 2.4 mM GSH and 1 mM CDNB (1-chloro-2,4-dinitrobenzene) as substrate [30]. Assay was initiated with 50 μl extract in 0.5 ml 0.1 M phosphate buffer, pH 6.5, at 25°C. One unit of enzyme activity is defined as the amount of enzyme catalyzing the oxidation of 1 mmol of substrate (CDNB)/ml/min at 25°C.

### Estimation of protein carbonyl (PC) content

The protein oxidation was monitored by measuring protein carbonyl (PC) content by using 2,4–dinitrophenyl hydrazine (DNPH) [31]. Briefly, 10% cold trichloroacetic acid (TCA) was added to the extract in 1∶1 ratio followed by centrifugation at 6000 g for 5 min at 4°C. 10 mM DNPH dissolved in 2 N HCl was added to the pellet and was allowed to stand in dark at RT for 1 h with intermittent vortexing. It was then centrifuged at 6000 g for 5 min at 4°C. The supernatant was discarded and 20% TCA was added to the pellet followed by centrifugation at 6000 g for 5 min at 4°C. The underivatized proteins in the pellet were washed 2–3 times with ethanol: ethyl acetate mixture (1∶1) till the pellet became clean. The final pellet was resuspended in 800 μl of 6 M guanidium hydrochloride and the absorbance was measured at 370 nm. The molar extinction of 22000×10^6^ μM^−1^cm^−1^ was used for calculations.

### Estimation of lipid peroxidation

The lipid peroxidation was estimated by the method of Okhawa *et al.* by measuring the malondialdehyde (MDA) level [32]. 100 μl of 10% SDS was added to 300 μl of extract and incubated for 5 min at RT. 600 μl of 20% acetic acid was added to the reaction mixture and incubated for 2 min at RT. Further, 600 μl 0.8% 2-thiobarbituric acid (TBA) was added and the final reaction volume was made up to 3 ml with distilled water. This was boiled for 1 h in water bath. The reaction mixture was allowed to cool at 4°C and then centrifuged at 10000 g for 5 min. The supernatant containing the active TBA reactive substances (TBARS) was collected and the absorbance was measured at 532 nm. The molar extinction coefficient of MDA used in the calculation was 1.53×10^5^ M^−1^ cm^−1^.

### NADPH oxidase activity assay

For NADPH oxidase activity, treated worms were homogenized in 50 mM phosphate buffer, pH 7.2 and 0.25% SDS. The homogenate was then centrifuged at 2000 rpm for 10 min at 4°C. 10 mM phosphate buffer, pH 7.2 containing 1 mM MgCl_2_, 80 μM cytochrome c and 2 mM sodium azide were added to 100 μl of supernatant obtained after centrifugation to make the final reaction volume to 1 ml. NADPH was added finally to initiate the reaction. The absorbance was read at 550 nm [33].

### Protein estimation

The protein was quantified in the extract by the method of Bradford using bovine serum albumin (BSA) as standard [34].

### Statistical analysis

All experiments were done in triplicate (n = 3) and the data were expressed as mean ± SEM. Mean, SEM and statistical significance were calculated using the GraphPad Prism 5.0. Statistical significance between the treated and the control worms was determined by using a two-tailed Student's t-test and P<0.05 was considered significant.

## Results

### Cytotoxic effect of E-64 on the *S. cervi* parasite

**Figure 1:**
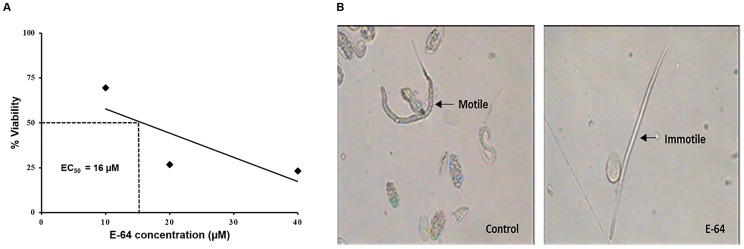
The effect of E-64 on viability of adult parasites and microfilariae. (A) Adult female worms (n = 10) of equal size were incubated in 20 ml maintenance at specified experimental conditions and viability was assessed by MTT assay after 8 h of exposure. Worms incubated in maintenance medium only served as control. (B) Microfilariae (mf) recovered from the uterus of gravid female of control and treated set. Adult female parasites were dissected longitudinally and the released mf was visualized under microscope at 40× (Motic B1 series). Data expressed is Mean ± SEM of at least three values (n = 3). ^***^P<0.0001, ^**^P<0.001, ^*^P<0.05. Values with P<0.05 were considered significant.

The *S. cervi* adult parasites were incubated in the KRB maintenance medium for 8 h at 37°C, 5% CO_2_ with 5, 10, 20 and 40 μM concentration of E-64. E-64 showed a concentration and time dependent decrease in motility and viability of the parasites as shown in Table 1 and Figure 1A respectively (EC_50_  =  16 μM). The microfilariae (mf) recovered from the uterus of gravid female parasites after 8 h were found to be motile up to 10 μM. They were under extreme stress and dead in case of 20 μM and 40 μM E-64 treated parasites at the end of 8 h (Figure 1B).

**Table 1:** Effect of E-64 on the motility of adult female *S. cervi* parasite.

Sample	Motility of parasites at following hours of incubation									Recovery©
	0 h	1 h	2 h	3 h	4 h	5 h	6 h	7 h	8 h	
Control	++++	++++	++++	++++	+++	+++	+++	+++	+++	++++
E-64 (5 μM)	++++	++++	++++	++++	+++	+++	+++	+++	++	+++
E-64 (10 μM)	++++	++++	++++	++++	+++	+++	++	++	++	+++
E-64 (20 μM)	++++	++++	+++	+++	++	++	+	+	-	-
E-64 (40 μM)	++++	+++	++	++	++	+	+	-	-	-

The motility of the parasites was visually checked at different time intervals. Adult female worms (n = 10) of equal size were incubated with 5, 10, 20 and 40 μM concentration of E-64 in 20 ml maintenance medium at 37°C and 5% CO_2_ for 8 h. Worms incubated in only maintenance medium served as Control. Worm motility was scored as -, no movement; +, least active; ++, less active; +++, moderately active; and ++++, highly active. ©Worms were transferred into fresh medium (devoid of E-64) after 8 h and motility recovery in treated group was compared to control group. Results are from three independent experiments performed in duplicates.

**Figure 2:**
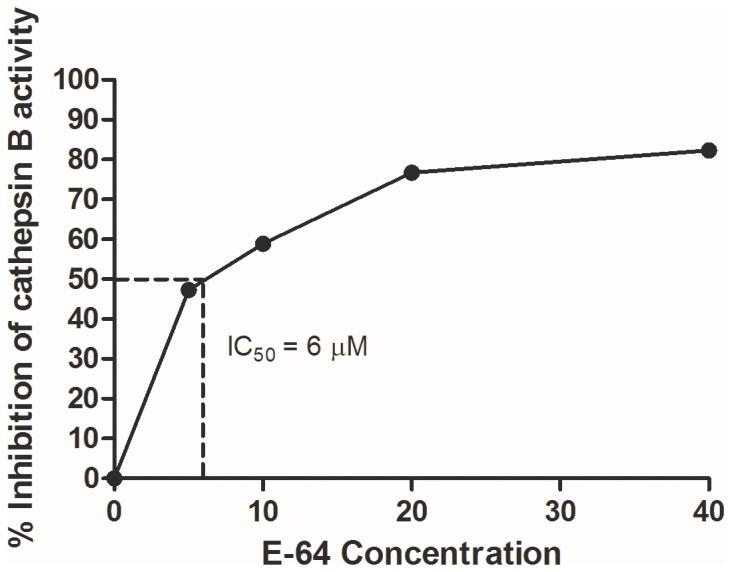
IC_50_ of E-64 for cathepsin B activity. The half maximal inhibitory concentration (IC_50_) was calculated by plotting the graph between the different concentration of E-64 and the % inhibition in cathepsin B activity.

The parasites exposed to various concentrations of E-64 showed concentration dependent significant decrease in cathepsin B activity after 8 h of treatment compared to the control parasites (Table 2). The IC_50_ of E-64 for cathepsin B activity was calculated to be 6 μM (Figure 2).

**Table 2:** Effect of E-64 on the activity of different enzymes in *S. cervi.*

	Control	E-64 (5 μM)	E-64 (10 μM)	E-64 (20 μM)	E-64 (40 μM)
Cathepsin B	111.76±6.039	58.89±0.66^***^	45.98±1.576^***^	26.07±1.007^***^	19.78±0.724^***^
		(−47.31%)	(−58.86%)	(−76.67%)	(−82.3%)
Caspase 3	9.43±0.266	10.02±0.199	13.76±0.404^***^	14.74±0.513^**^	15.72±0.689^**^
		(+6.25%)	(+46.0%)	(+56.36%)	(+66.8%)
Glutathione-S-transferase	24.30±0.567	20.46±1.267	19.20±0.337^**^	18.03±0.275^***^	14.73±0.482^***^
		(−15.82%)	(−21.0%)	(−25.8%)	(−39.40%)
Glutathione reductase	19.20±1.228	15.36±1.784	14.02±1.618	9.93±0.902^**^	7.97±0.786^**^
		(−20.0%)	(−27.0%)	(−48.26%)	(−58.5%)
Protein Tyrosine Phosphatases	127.84±0.624	106.84±0.563^***^	93.07±0.912^***^	73.21±1.088^***^	35.03±0.938^***^
		(−16.27%)	(−27.20%)	(−42.73%)	(−72.6%)
Cytochrome c oxidase	2.445±0.192	2.181±0.073	1.86±2.442^***^	1.49±1.127^***^	0.96±0.52^***^
		(−10.78%)	(−24.0%)	(−39.13%)	(−60.9%)

Adult female worms (n = 10) were incubated with different concentration of E-64 separately in 20 ml maintenance medium at 37°C. Worms incubated in only maintenance medium served as Control. After 8 h of incubation different enzyme activity were checked according to the method given in materials and methods section. Activities are indicated as U/ml. Values in parentheses indicate percentage inhibition/activation in comparison to control. Data expressed is Mean ± SEM of at least three values (n = 3). ^***^P<0.0001, ^**^P<0.001, ^*^P<0.05. Values with P<0.05 were considered significant.

**Figure 3:**
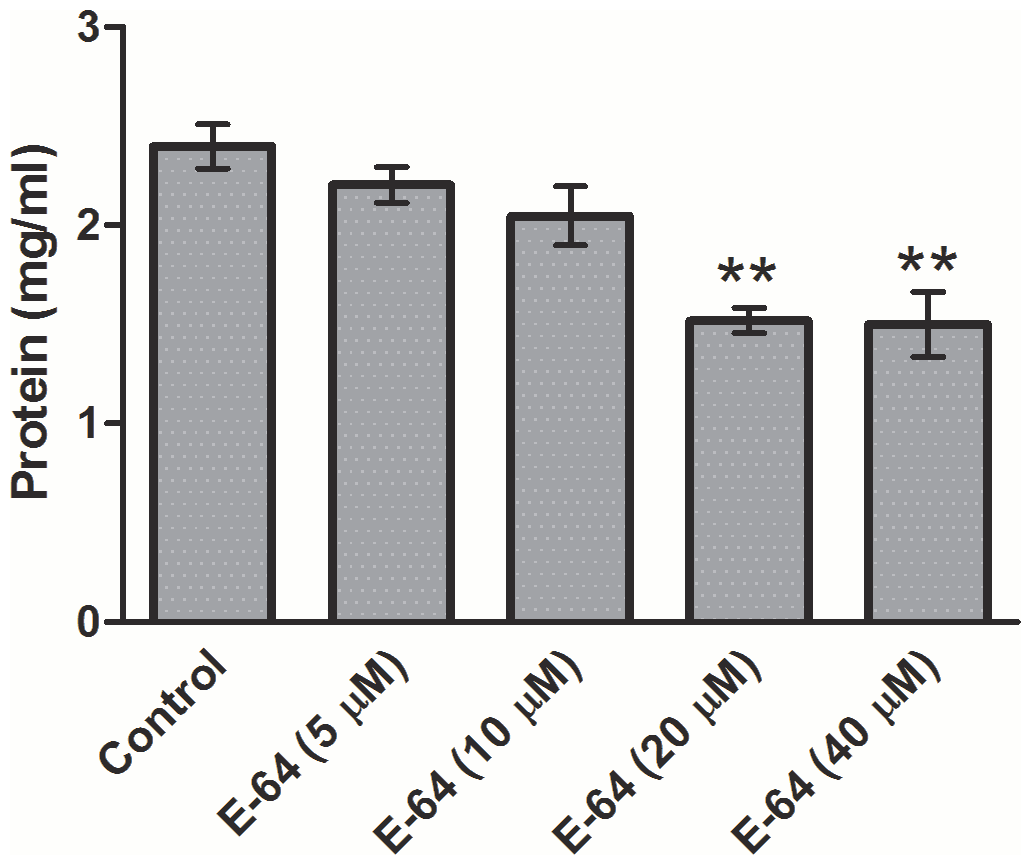
The protein content of the control and E-64 treated parasites. The protein content was determined using Bradford assay using BSA as standard. Data expressed is Mean ± SEM of at least three values (n = 3). ^***^P<0.0001, ^**^P<0.001, ^*^P<0.05. Values with P<0.05 were considered significant.

A significant decrease in the protein level was observed in E-64 treated parasites as compared to control (Figure 3).

### Effect of E-64 on different apoptotic markers in the parasite

**Figure 4:**
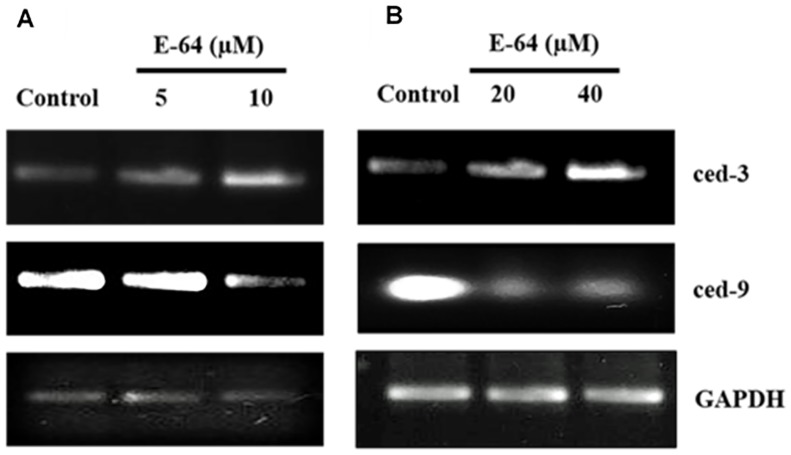
The expression of *ced-3* and *ced-9* in the control and parasites exposed to E-64. GAPDH was used as a loading control in the experiment.

The levels of apoptotic markers like expression of ced-9, ced-3 and activity of cytochrome c oxidase, CED-3/caspase 3 and tyrosine phosphatases were evaluated in the control and E-64 treated sets after 8 h of treatment. The ced-3 and ced-9 expression was up and down regulated respectively in a concentration dependent manner in the parasites exposed to E-64 after RT-PCR (Figure 4). An increase in the biochemical activity of caspase 3/CED-3 in treated parasites was also observed. The cytochrome c oxidase activity associated with the inner mitochondrial membrane showed a decline upto 60.9% at 40 μM E-64 concentration. The protein tyrosine phosphatases (PTP) which are known to induce apoptosis indirectly were also significantly inhibited upto 73% in the parasites exposed to E-64 at 40 μM concentration (Table 2).

### Effect of E-64 on various oxidative stress parameters in the treated parasites

**Figure 5:**
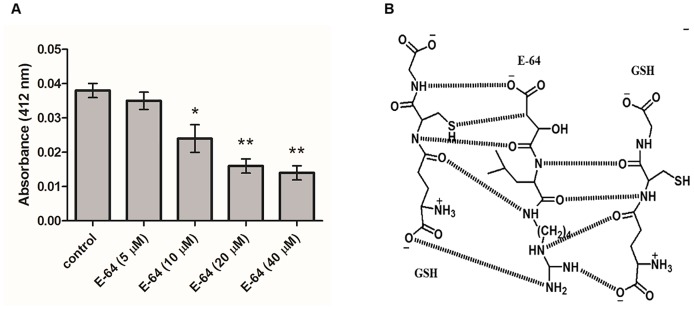
Interaction of E-64 with GSH was drawn using ChemDraw Ultra 7.0. (A) *In vitro* effect of E-64 on GSH using DTNB as substrate (B) Possible interaction of E-64 with GSH molecule.

Various oxidative stress parameters were evaluated in the E-64 treated parasites after 8 h. Depletion in the glutathione (GSH) level was found in the parasites incubated with 5, 10, 20 and 40 μM E-64 as compared to the control parasites (Table 3). To find out if E-64 has any direct effect on GSH, an *in vitro* study was performed by incubating different concentration of E-64 with 10 μM GSH. There was a significant reduction of 63% in absorbance (412 nm) at 40 μM E-64 (Figure 5A). Also, a possible interaction between E-64 and GSH has been shown (Figure 5B). The structures and interactions were drawn with the help of ChemDraw Ultra 7.0. There was a concentration dependent decrease in the activities of antioxidant enzymes GR and GST (Table 3). E-64 treated parasites also showed a concentration dependent increase in NADPH oxidase activity (Table 3).

**Table 3:** Effect of E-64 on different oxidative stress markers in *S. cervi*.

Samples	GSH	Protein Carbonyl content	Lipid peroxidation	NADPH oxidase
Control	3.705±0.294	29.09±1.353	14.50±1.524	0.489±0.043
E-64 (5 μM)	3.27±0.387	31.95±2.346^**^	15.66±0.858	0.583±0.029
	(−11.74%)	(+9.83%)	(1.08 fold increase)	(+19.26%)
E-64 (10 μM)	3.12±0.112	32.697±0.676	19.865±0.08^*^	0.616±0.009^*^
	(−15.8%)	(+12.4%)	(1.37 fold increase)	(+25.97%)
E-64 (20 μM)	2.607±0.094^*^	34.507±1.784	28.275±0.023^***^	0.852±0.035^**^
	(−29.63%)	(+18.62%)	(1.98 fold increase)	(+74.23%)
E-64 (40 μM)	1.978±0.100^**^	38.602±0.622^**^	33.06±0.181^***^	0.894±0.009^***^
	(−46.61%)	(+32.7%)	(2.16 fold increase)	(+82.82%)

Adult female worms (n = 10) were incubated with different concentration of E-64 separately in 20 ml maintenance medium at 37°C. Worms incubated in only maintenance medium served as Control. After 8 h of incubation different enzyme activity were checked according to the method given in materials and methods section. The level of GSH is expressed as μM/mg protein. Protein carbonyl content is expressed in terms of μmol/mg protein. The level of lipid peroxidation is expressed in terms of μmol MDA/mg protein. NADPH oxidase activity is expressed as U/ml. Values in parentheses indicate percentage inhibition/activation in comparison to control. Data expressed is Mean ± SEM of at least three values (n = 3). ^***^P<0.0001, ^**^P<0.001, ^*^P<0.05. Values with P<0.05 were considered significant.

**Figure 6:**
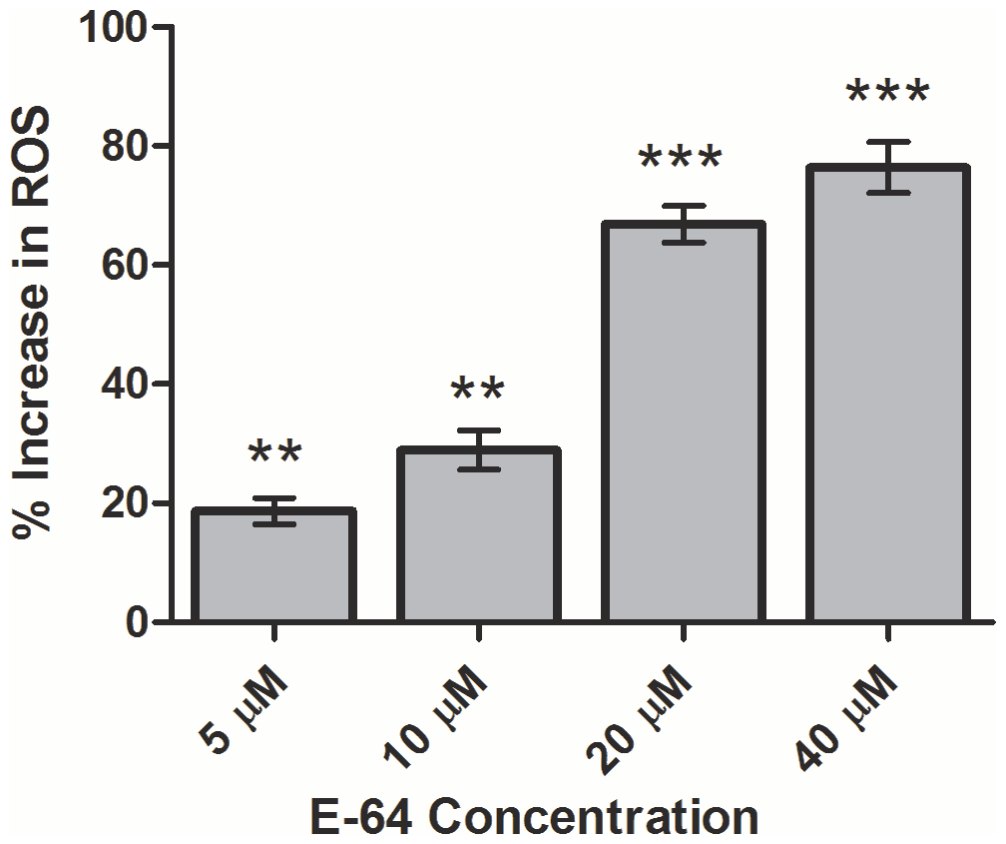
The effect of E-64 on total reactive oxygen species production in parasites. Adult female parasites were exposed to 5, 10, 20 and 40 μM E-64 for 8 h in the KRB maintenance medium. Total Reactive oxygen species generated was measured using NBT as substrate. The data expressed is Mean ± SEM of at least three values (n = 3). ^***^P<0.0001, ^**^P<0.001, ^*^P<0.05. Values with P<0.05 were considered significant.

A significant increase in ROS generation was observed in E-64 treated worms as compared to the control parasites (Figure 6). Lipid peroxidation and protein oxidation were measured in terms of MDA and PC levels in the treated parasites. The protein carbonyl levels were increased on exposure to 5, 10, 20 and 40 μM E-64 as compared with control parasites. The MDA level was significantly increased in parasites exposed to 10, 20 and 40 μM E-64 as compared to the control parasites (Table 3).

## Discussion

Effective chemotherapy for the lymphatic filariasis is lacking till date. The antifilarial drugs recommended by Mass drug administration only kill larval stages of the parasite without affecting the adults [35]. This problem makes it essential to look for specific drug targets in the filarial worms crucial for their survival. Parasitic helminths are known to possess cathepsins which encompass many functions that facilitate parasitism including migration, immune evasion, excystment, and feeding. Hence parasitologists are very ardent to work on this family of cysteine proteases [36]. Their role is well documented in case of nematodes. Recently, a cathepsin B like cysteine protease was, expressed in early juvenile stages of *Fasciola gigantica*, and used as a vaccine candidate to block the invasion and migration of parasites [37]. Thereby suggesting that the inactivation of cathepsin B like cysteine proteases either by vaccination or chemotherapy would significantly reduce the damage caused by these parasitic helminths to the host tissues. These observations encouraged us to explore the role of these proteases in filarial parasites too. We have used E-64, a cathepsin B cysteine protease inhibitor for our studies. The mechanism how E-64 inhibits cysteine proteases has been described earlier [12], [13]. The inhibitor interacts with the enzyme through the formation of a covalent bond between its epoxy carbon atom and the Cys-SH residue at the active site of the cysteine proteases. This is a crucial step for the inhibitory action of E-64. Apart from cysteine, the amino acids like glycine and glutamate form weak interaction with E-64 and help in stabilizing the enzyme inhibitor complex. The present manuscript reports that E-64 inhibits filarial cathepsin B in *ex vivo* conditions reducing motility and viability of the parasites. It further leads to induction of oxidative stress followed by mitochondrial mediated apoptosis in filarial parasites.

E-64 reduced the motility of filarial parasites in a time and concentration dependent manner. While the parasites in the 5 and 10 μM treatment set recovered when transferred to the fresh medium, the sets exposed to 20 and 40 μM concentration did not show recovery and were completely immotile after 8 h. The viability of the parasites also showed a dose dependent reduction. The EC_50_ for viability of the adult parasites was calculated to be 16 μM. In addition, the cathepsin B activity in the treated parasites was also recorded at the end of 8 h. The IC_50_ calculated for the for the cathepsin B activity was found to be 6 μM which is lower than that reported in case of human (10–20 μM) exhibiting that E-64 is more specific and effective against filarial parasites than the human host.

As it is reported that E-64 causes apoptosis making the lysosomal proteolytic pathway antagonist to the cell survival pathway in human neuroblastoma cells [38]. We, therefore evaluated different apoptotic parameters like expression of ced-3 and ced-9, activity of caspase 3, protein tyrosine phosphatases and cytochrome c oxidase in the treated parasites. The pro- and anti-apoptotic genes required for programmed cell death during the development of nematode *Caenorhabditis elegans* has been reported earlier. The ced-9 is an anti-apoptotic gene encoding homolog of the mammalian cell-death inhibitor Bcl-2. Its activity is vital for preventing cells from undergoing programmed cell death. The ced-3 is a pro-apoptotic gene encoding a protein that resembles mammalian interleukin-1β converting enzyme i.e. caspase 3, which is a cysteinyl aspartate protease regulating programmed cell death in worms [39]. CED-3 activity is important for execution of apoptosis and functions in a conserved genetic pathway with CED-4, CED-9, and EGL-1 to regulate apoptosis during *C. elegans* development. The results of our study shows that E-64 downregulates ced-9 and upregulates ced-3 expression and in a concentration dependent manner. The apoptotic event initiated in the filarial parasites exposed to E-64 at 10 μM is evident from the decreased expression of ced-9 followed by elevation in the ced-3 expression/caspase 3 activity in parasites exposed to further higher inhibitor concentration. The observation of our results is supported by the finding in *C. elegans* which states that ced-9 function lies upstream to ced-3 expression facilitating the proteolytic cleavage of inactive pro-CED-3 to active caspase 3 activity finally executing cell death event [40]. However, a significant inhibition in the cathepsin B activity occurred at 5 μM concentration before any significant alterations in the apoptotic markers started. A report by Malla *et al.* also showed that it is the inhibition of cathepsin B first which led to upregulation of BAX/Bcl2 ratio leading to apoptosis in human glioma cells [11]. Loss in the mitochondrial membrane integrity resulting in the release of cytochrome c in the cytosol is another event associated with mitochondrial mediated apoptosis [41]. We also found a decrease in the cytochrome c oxidase activity suggesting disruption of mitochondrial membrane. Cytochrome-c forms a multimeric complex with apoptotic activating factor (Apaf-1) leading to activation of CED-3/caspase 3, cysteinyl aspartate-specific proteases, as also shown by our studies in the treated parasites. It is also reported that E-64, being a cysteine protease inhibitor is incapable of inhibiting caspase 3 belonging to cysteinyl aspartic family of proteases [42]. Caspase 3 is a crucial molecule for executing both the intrinsic and extrinsic apoptotic pathway.

Exposure of E-64 also resulted in inhibition of the tyrosine phosphatase activity in treated parasites. The decrease in PTPs indicates an increased phosphorylation of proteins as well as in the level of MAPKs. The MAPKs are regulated through reversible phosphorylation of tyrosine and threonine in their thr-xaa-tyr motifs. It has also been stated that there is loss in the anti-apoptotic activity of Bcl2 when it is in phosphorylated form which is due to decrease in tyrosine phosphatase activity, leading to cell death. It has also been shown that PTPs play an important role in the regulation of the apoptotic genes of Bcl-2 family, directly inducing the expression of Bax and pro-apoptotic proteins and thus leading to apoptosis [43].

Filarial parasites have a long life span and constantly face stress conditions within the host. They have evolved a series of enzymatic and non-enzymatic antioxidant system in order to evade the host's hostile environment [44]. As mentioned earlier that apoptosis is triggered by oxidative damage, we checked different oxidative stress parameters to find out the exact mechanism of induction of apoptosis in these parasites. Glutathione (GSH) is one of the key antioxidant molecules in the filarial parasites. It regulates the intracellular redox homeostasis by the reversible oxidation of active thiol groups of various proteins [45], [46]. GSH also acts as a free radical and electrophile xenobiotic scavenger. Enzymatic antioxidants such as GST and GR acts as a first line of defence and maintain cellular redox system by scavenging the free radicals. GST is the major detoxification enzyme in the filarial parasites as they lack cytochrome P-450 system. It is a GSH requiring enzyme and is mainly involved in detoxification of electrophilic compounds [47]. Glutathione reductase is another antioxidant flavoenzyme that reduces oxidized glutathione (GSSG) to reduced glutathione (GSH) and maintains high ratio of [GSH]/[GSSG] within the parasite [16]. Thus, protecting the parasite from the lethal effect of the reactive oxygen species produced within the host. Interestingly, the E-64 exposure significantly decreased the activity of major antioxidant enzymes as well as GSH. We have also observed a significant decrease in the parasitic GR and GST levels in the E-64 exposed worms. How E-64 is affecting antioxidant system is not clear as there are no reports available. However, we have checked the direct effect of E-64 on GSH *in vitro* and found significant decrease in GSH level as compared to the control set. It has been observed that in the presence of DTNB, there was percentage reduction in absorbance with increasing concentration of GSH as compared to control set. Structurally, GSH, a γ-glutamylcysteine glycine tripeptide resembles the amino acid alignment of cysteine proteases participating in the interaction with E-64. There may be a possibility that the Cys residue of the GSH might form covalent bond with E-64 epoxy group. However, Gly and Glu residues may participate in other weak interactions with E-64 in a similar way as that of cysteine proteases. Thus speculating that, E-64 being specific inhibitor of cysteine proteases might render GSH availability as an antioxidant to the parasite.

Reactive oxygen species are continuously produced as a by-product of cellular metabolism under normal physiological conditions and are scavenged by cellular defence system. Therefore, maintaining a balance between ROS and antioxidant defence system. However, during stress conditions, diminution of antioxidant system leads to enhanced ROS generation leading to oxidative stress. In our present investigation, an increase in the reactive oxygen species level and NADPH oxidase activity was observed in the E-64 treated parasites. The activated NADPH oxidase transports electrons from NADPH on the cytoplasmic side of the membrane to oxygen in the extracellular fluid to form (O_2_^•-^) [48]. Through spontaneous reactions superoxide anion could further lead to the formation of hydrogen peroxide (H_2_O_2_) and other reactive oxygen species.

It is well known that reactive oxygen species cause damage to the mitochondrial membrane lipids, further leading to the disturbance of membrane organization, and production of secondary lipid peroxidation products. The present study also showed elevated levels of lipid peroxidation products as high as 128% above basal values in parasites after E-64 exposure. The reactive oxygen species generated could also lead to the protein oxidation which is evidenced by the marked increase in the protein carbonyl content in the treated parasites.

Oxidative stress may also lead to the oxidation of cysteine thiol containing enzymes such as protein tyrosine phosphatases and in turn cause their inhibition [49]. It has been reported that the oxidative stress generated in scleroderma dermal fibroblasts leads to suppression in the expression of PTP1B level [50]. Reactive oxygen species are known inducers of mitochondrial mediated apoptosis and act by increasing the mitochondrial membrane permeability through Bax-Bcl2 interaction. Also, an increase in lipid peroxidation in the mitochondrial membrane might lead to decrease in the activity of cytochrome c oxidase [41].

In conclusion, our study explains that E-64 significantly inhibits filarial cathepsin B activity followed by generation of oxidative stress and induction of a mitochondrial mediated apoptotic like event in filarial parasites. Thus, exhibiting the important role played by these filarial cathepsin B in the survival of these parasites. Thus suggesting the use of filarial cathepsin B like cysteine proteases as a good chemotherapeutic target for lymphatic filariasis.
